# Complementary Deep and Shallow Learning with Boosting for Public Transportation Safety

**DOI:** 10.3390/s20174671

**Published:** 2020-08-19

**Authors:** Shengda Luo, Alex Po Leung, Xingzhao Qiu, Jan Y. K. Chan, Haozhi Huang

**Affiliations:** 1 Faculty of Information Technology, Macau University of Science and Technology, Taipa 999078, Macao; shengdaluo1991@gmail.com (S.L.); xingzhaoq@student.unimelb.edu.au (X.Q.); 1509852hii30001@student.must.edu.mo (J.Y.K.C.); haozhihuang@fudan-zhuhai.org.cn (H.H.); 2 Melbourne School of Engineering, the University of Melbourne, Melbourne 3010, Australia; 3 The School of Computer Science, Fudan University, Shanghai 201203, China; 4 Zhuhai Fudan Innovation Institute, Zhuhai 519000, China

**Keywords:** controller area network, transportation, deep learning, machine learning

## Abstract

To monitor road safety, billions of records can be generated by Controller Area Network bus each day on public transportation. Automation to determine whether certain driving behaviour of drivers on public transportation can be considered safe on the road using artificial intelligence or machine learning techniques for big data analytics has become a possibility recently. Due to the high false classification rates of the current methods, our goal is to build a practical and accurate method for road safety predictions that automatically determine if the driving behaviour is safe on public transportation. In this paper, our main contributions include (1) a novel feature extraction method because of the lack of informative features in raw CAN bus data, (2) a novel boosting method for driving behaviour classification (safe or unsafe) to combine advantages of deep learning and shallow learning methods with much improved performance, and (3) an evaluation of our method using a real-world data to provide accurate labels from domain experts in the public transportation industry for the first time. The experiments show that the proposed boosting method with our proposed features outperforms seven other popular methods on the real-world dataset by 5.9% and 5.5%.

## 1. Introduction

### 1.1. Motivations

Decreasing the current number of global deaths and injuries from road traffic accidents by half is one of the important Sustainable Development Goals as part of the 2030 Agenda for Sustainable Development adopted by the United Nations General Assembly. Traffic accidents not only bring huge financial losses to society but also cause great physical and mental damages to everyone [1,2]. Millions of people died from traffic accidents in 2018 [3], and most traffic accidents are caused by human mishandling [4]. Analyzing the behavior of drivers, especially public transportation drivers, is important to protect road safety [5,6,7]. To ensure safety for public transportation, public transportation operators can be requested to get an evaluation of drivers and to identify dangerous drivers for retraining. For public transportation fleet management and monitoring, massive data is collected from vehicles using state-of-the-art technologies of sensors for example MobilEye from Israel. In the control center, thousands of real-time events and alarms arrive from the sensors of the vehicles through wireless networks in real time every day. Although it is virtually impossible to handle such a huge amount of data manually, accurate predictions with machine learning to analyze behavior from the vehicles has recently become feasible. Machine learning techniques have been applied to analyzing behavior in different tasks with various kinds of data collected using sensors in moving vehicles [8,9,10,11]. We investigate efficient and accurate machine learning methods to classify whether the driving behavior of drivers is safe as it is important that drivers with unsafe behavior should be warned and retraining can be provided to the drivers.

### 1.2. Challenges

It is challenging to classify a driver’s driving behavior (safe/unsafe). First, the industrial need for a high classification performance cannot be satisfied using solely existing methods as the misclassification rates can be high. The high misclassification rates in previous methods have been pointed out in previous literature  [12,13] as it has been suggested that it is difficult to model the driver’s behavior. For example, the performance of K Nearest Neighbour (KNN) models is greatly affected by unbalanced training data in traffic data for safety with many fewer labels for accidents. Second, the lack of features in the data collected with the Controller Area Network (CAN) bus does not provide lots of information for driving behavior analysis to train an accurate machine learning classifier. There is no existing method for road safety predictions with CAN bus data to extract extra useful information from features. Last, because of the high cost of labeling and privacy issues with public transportation data, to the best of our knowledge, there is no publicly available dataset with labels for the predictions of safe driving behavior on public transportation. The lack of urgently needed labels in datasets for road safety makes it hard to evaluate and build machine learning models.

### 1.3. Contributions

In this paper, our contributions include (1) a boosting method to make deep learning and statistical learning complement each other, (2) a novel method to compute extra time-series features to extract richer information, (3) extensive evaluation on a new real-world dataset with labels from experts in the public transportation domain.

The motivation for the usage of CAN-bus data is that the CAN standard is one of the most important bus standards for vehicles [14,15,16,17]. The infrastructure already built-in in most vehicles bought by transportation companies records data efficiently from devices in the vehicles. The CAN standard is a serial data bus standard designed to enable electronic devices to communicate with each other. Therefore, CAN data can be easily obtained by transportation companies. There are a small number of available features in the data collected using the standard CAN bus system. With the lack of sufficient useful features, it is hard to find patterns in the data to determine driving behavior from the driver.

The novel boosting method is proposed to combine the advantages of statistical learning and deep learning. It is shown in our experiments that the ensemble with our proposed features outperforms any single state-of-the-art method we considered, and our boosting method combines seven state-of-the-art machine learning methods including support vector machine (SVM), random forest (RF), *k*-nearest neighbour (KNN), discriminant analysis, naive Bayes classifier, adaptive boosting (AdaBoost) and a deep learning neural network called Long Short-Term Memory (LSTM). In addition, the proposed boosting method outperforms the seven methods also for the case without the proposed feature extraction method.

Extra features are computed using our method. Feature engineering is an important tool to extract useful information in time-series for machine learning methods for better performance when the number of features is small. We show in our experiments that this significantly improves the performance of the classifiers. We, thus, propose a method to compute extra time-series features from the raw data of the CAN bus system to extract extra information.

To completely evaluate methods in the real world, the experiments are conducted using a real-world dataset collected using the CAN bus system. Because of the high cost of sample labeling, there is no published real-world dataset with labels for analyzing the driving behavior of drivers on public transportation. The samples in the dataset are labeled by the experts of Transportes Urbanos de Macau (TransMac), which is one of the largest bus companies in Macao.

We divide the rest of this paper into five sections. In Section 2, we describe related work. Our dataset and the proposed method are described in detail in Section 3 and Section 4. In Section 5, the proposed method is evaluated on two datasets with a comparison to various state-of-the-art machine learning methods. Section 6 concludes this work.

## 2. Related Work

In this work, we focus on safety classification with data from the Controller Area Network (CAN) bus. The development of the CAN bus started in 1983 and was released in 1986 [18]. This standard has recently become available in most embedded systems for vehicles. The CAN bus is one of five protocols used in the mandatory onboard diagnostic (OBD-II) standard. The OBD-II standard has been mandatory for all cars and light trucks sold in the United States since 1996, and the EOBD standard has been mandatory for all petrol vehicles sold in the European Union since 2001 and all diesel vehicles since 2004 [19,20]. A lot of recent research focuses on the analysis of sensor data from the CAN bus system [5,21,22,23,24,25,26,27,28].

Machine learning plays the important role in building data analytics models to handle massive data [29,30]. Statistical learning and deep learning are important areas in machine learning. Methods based on statistical learning have been successfully applied to solving many related behavior analytics problems. In [11,31], Bayesian methods are used to predict braking behavior and model vehicle speeds. *k*-nearest neighbors (KNN) is employed to classify driving styles in [10]. Support vector machines (SVM), deep learning (DL), and decision trees (DT) have been applied to predicting driving behavior and accident risk predictions [8,9]. Deep learning techniques are popular in recent years for many tasks [32,33]. The Long Short-Term Memory (LSTM) technique is one of the most popular deep learning techniques for time-series problems [34]. LSTM networks are a special kind of recurrent neural networks (RNN), and LSTM networks aim at learning long-term dependencies [35]. In contrast to the standard RNN, the repeating modules of LSTM networks contain four interacting layers to enable the ability to change information of the cell state. LSTM is used to detect driver distraction [36]. The ensemble of machine learning methods are shown to outperform, most of the time, one particular technique [37,38,39], and we propose a heterogeneous boosting method to obtain better performance. In our experiments, our boosting method is compared with seven state-of-the-art methods.

## 3. Evaluation of Road Safety Predictions

For any application domain of machine learning, one of the most objective evaluation methods is to see how prediction models perform in real-world datasets. However, to the best of our knowledge, there is no published real-world dataset with labels provided for behavior analysis of public drivers. In this work, we build a new dataset collected using the CAN bus system from one of two public bus companies in Macao called TransMac.

One record is produced every three seconds from the reading of the sensors in a moving public vehicle, and there are totally 6451 records with 24 features in the new dataset. All 6451 records are labeled by domain experts. There are 507 unsafe cases and 5944 safe cases in the labels. In total, the recording time for our CAN bus data is 6451×3= 19,353 s which is 5.38 h of driving by professional bus drivers in the company. Although each sample contains 24 features, some features are totally irrelevant for the training of the machine learning model (see Table 1). As shown in Table 1, features like vehicle identification are meaningless for the machine learning model. Features containing too much missing data cannot be useful either. For example, most entries of the “CANALARMSTATE” feature and the “CANALARMSTATE” are N/As (Not Available). Features used are listed in Table 1 to provide a reference for the training of our method. In addition, descriptive statistics on the feature set used for training are shown in Table 2.

## 4. The Proposed Method

We propose a feature extraction method (see Algorithm 1) for extracting richer information from the change of feature vectors against time and propose a boosting method (see Algorithm 2) to classify whether driving behavior of drivers on public transportation can be considered safe on the road. The feature extraction method is a general method. It can be used with any other machine learning classification method. In the experiments, it is shown that our feature extraction method can be used to improve the performance of any classification method. In addition, it is also shown that our boosting method outperforms other seven machine learning methods whether or not our feature extraction method is used.

### 4.1. Our Method for Richer Information With Feature Extraction

Missing data is common in industrial data collected from CAN data systems. Features with too many N/A (Not Available) entries cannot be used to train the machine learning methods. In addition, features irrelevant for driving behavior analysis like the identifier of the vehicles are excluded. Therefore, there are only a few useful features left for classification without irrelevant features (see Table 1). The low dimensionality of the feature space of training data severely limits the descriptive power of the samples. The lack of descriptive power makes it is very difficult to obtain accurate machine learning models. We argue that richer information can be extracted from the change of feature values against time and we, hence, propose a feature extraction method to provide extra useful time-series features to deal with the lack of information in the original features. For example, the acceleration of the car is important for driver behavior analysis, but this information is not recorded in the original data. The acceleration of the bus can be obtained by calculating the gradient of the velocity of the bus. The proposed feature extraction method is shown in Algorithm 1.
**Algorithm 1** Our Method for Richer Information with Feature Extraction**Input:***n* samples $\left\{s_{1},\ldots ,s_{n}\right\}$ where $s_{i}={\left[f_{i,1},\ldots ,f_{i,m}\right]}^{T}$**Output:***n* samples $\left\{s_{1},\ldots ,s_{n}\right\}$ with time-series features, $s_{i}={\left[f_{i,1},\ldots ,f_{i,m},t_{i,1},\ldots ,t_{i,m+7}\right]}^{T}$  Divide *n* samples into *p* periods, $\left\{P_{1},\ldots ,P_{p}\right\}$, by the recording time.  **for**
j=1,…,p
**do**   **for** each sample $s_{i}\in P_{j}$
**do**    ${\left[t_{i,1},\ldots ,t_{i,m}\right]}^{T}=\frac{1}{\left|P_{j}\right|}\times \sum_{s_{z}\in P_{j}}{\left[f_{z,1},\ldots ,f_{z,m}\right]}^{T}$    $s_{i}={\left[f_{i,1},\ldots ,f_{i,m},t_{i,1},\ldots ,t_{i,m}\right]}^{T}$   **end for**  **end for**  **for** each sample $s_{i}$
**do**   $t_{i,m+1}$ and $t_{i,m+2}$ are the differences in the feature values of $s_{i}$, for the latitude and the longitude respectively.   $\left\{t_{i,m+3},\ldots ,t_{i,m+7}\right\}$ are the gradients of the feature values of $s_{i}$ related to the velocity, the mileage,   the tire pressure, the engine speed, and the engine temperature.   $s_{i}={\left[f_{i,1},\ldots ,f_{i,m},t_{i,1},\ldots ,t_{i,m+7}\right]}^{T}$  **end for**

The input data of Algorithm 1 contains *n* samples, $\left\{s_{1},\ldots ,s_{n}\right\}$, with *m* features $s_{i}={\left[f_{i,1},\ldots ,f_{i,m}\right]}^{T}$. Especially, the features irrelevant for training are excluded in these *m* features. For example, for our proposed dataset, the *m* features are the twelve features used to train the classification model (see Table 1). It is common in time-series analysis to use moving averages. The moving average is used to filter out noise. It is a common signal preprocessing step for time-series data if there is noise in the data [40,41,42]. We noticed the noise in the CAN-bus data so signal processing filtering techniques are employed in our work with the aim to achieve better training and classification. The moving average is used to filter out noise. It is a common signal preprocessing step for time-series data if there is noise in the data [40,41,42]. We noticed noise in the CAN-bus data so signal processing filtering techniques are employed in our work with the aim to achieve better training and classification.

For example, the average driving speed of a driver in two minutes (a period) is useful information for analyzing his driving behavior. Motivated by this, some time-series features are calculated for this particular reason (see Algorithm 1). The *n* samples are divided into *p* periods by the recording time. The value of *p* is a tunable parameter, and it depends on the time interval between two samples.

In our boosting method, one period covers two minutes. For sample $s_{i}$, *m* time-series features, $\left\{t_{i,1},\ldots ,t_{i,m}\right\}$, are extracted from raw features. The latitude and longitude features, $f_{latitude}$ and $f_{longitude}$, are obtained from GPS information. The difference in the latitude/longitude values of the adjacent samples can be used to measure the velocity of the bus. In Algorithm 1, $t_{i,m+1}$ and $t_{i,m+2}$ of sample $s_{i}$ are the differences in the values of sample $s_{i}$ and sample $s_{i-1}$, for latitude and longitude features respectively. The gradient of a feature is used to describe how fast the feature values change. The velocity, the mileage, the tire pressure, the engine speed, and the engine temperature are important for accurate classification. These features can reflect the different behavior of drivers, and $\left\{t_{m+3},\ldots ,t_{m+7}\right\}$ are calculated to find the rates of change.

The features are irrelevant features if the changes of the features due to reasons other than driving behavior. In most cases, data used to train machine learning models includes irrelevant features or redundant features. Certain machine learning models automatically pick useful features during training. We use techniques that specifically map useful features to the labels (driving behavior in our case) effectively like random forests (RF) with the importance scores of features generated to ignore irrelevant features. The RF model is then trained using a subset of features with high importance scores [43,44,45]. When the dimensionality of feature space is large, performance could suffer due to the curse of dimensionality [46,47]. However, in our case, as the number of features is not exceedingly high, our experiments show that the techniques perform well without suffering from the curse of dimensionality.

### 4.2. Weak Learners

In our heterogeneous boosting method, all the seven methods in Table 3 are used. Different machine learning methods have their own characteristics and are suitable for different tasks. SVM, traditionally a statistical machine learning method, is one of the most popular methods for two-class classification. In SVM, a hyperplane is constructed for classification after the data in the low-dimensional space is mapped to the high-dimensional space using a kernel function. In our boosting methods, Radial Basis Function (RBF) kernel is used as the kernel function of the SVM, and the RBF kernel can be obtained as

(1) $$R\left(x_{i},x_{j}\right)=exp\left(-\gamma {∥x_{i}-x_{j}∥}^{2}\right),$$

where $x_{i}$, $x_{j}$ are the feature vectors of sample *i* and sample *j*. *k*-nearest-neighbors (KNN) is a nonparametric classification method, which is simple but effective in machine learning. The assumption underlying KNN is that the information (e.g., the class in classification problems) of an output sample is similar to input samples containing similar characteristics to this output sample. In the proposed boosting method, KNN using the chi-square distance is used to measure the distance of samples. The chi-square distance between sample *i* and sample *j* is obtained as

(2) $$\chi \left(x_{i},x_{j}\right)=\sqrt{\sum_{f=1}^{F}w_{f}{\left(x_{i,f},x_{j,f}\right)}^{2}},$$

where $x_{i}$, $x_{j}$ are the feature vectors, and *F* is the dimentionality of samples. RF, a method based on bagging, contains a certain number of decision trees to classify. In the RF method, *Q* decision trees are trained to determine classification results of RF. Given a sample *x*, its result *y* produced by RF is

(3) $$y=\frac{1}{Q}\sum_{q=1}^{Q}f_{q}\left(x\right),$$

where $f_{q}\left(x\right)$ is the result given by a decision tree. Discriminant Analysis is also known as Fisher Discriminant Analysis. In Discriminant Analysis, a linear combination of the features is obtained to classify the samples. AdaBoost used as one of the component learners in our boosting method is a homogeneous boosting method. The base learners of this homogeneous boosting method are 1-depth decision trees. In Naive Bayes, Bayes’ theorem with naive independence assumptions between the features is applied.

**Figure 1:**
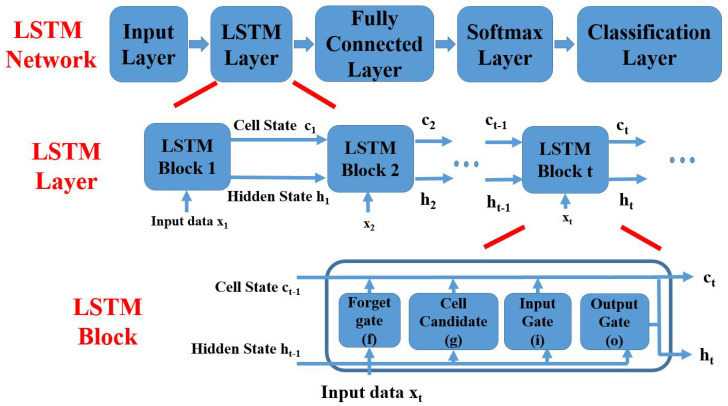
The long short-term memory (LSTM) network used in the proposed boosting method.

The LSTM network used in the proposed boosting method is shown in Figure 1. There are five layers in the LSTM network: the input layer, the LSTM layer, the fully connected layer, the softmax layer, and the classification layer. The LSTM network starts with putting the CAN bus data into the input layer. Using the input data as the training set, the LSTM layer learns long-term dependencies of samples. To handle time-series data, the LSTM layer contains many LSTM blocks. One LSTM block uses an input sample and the output of last LSTM block as its input and the block output a cell state and a hidden state. Finally, the classification results are generated by the fully connected layer, the softmax layer, and the classification layer through analyzing the long-term dependencies. In the LSTM network, the vanishing gradient problem is avoided by adding four components to the RNN network (see Table 4 and Figure 1): the input gate, the output gate, the forget gate, and the cell candidate. The interaction of the four components is shown in Table 4 and Figure 1. $c_{t}$ and $h_{t}$ denote the cell state and the hidden state produced by the *t*-th LSTM block. $i_{t}$, $o_{t}$, $f_{t}$, and $c_{t}$ denote the outputs of the input gate, the output gate, the forget gate, and the cell candidate. As shown in Figure 1, the output of the *t*-th LSTM block are the cell state $c_{t}$ and the hidden state $h_{t}$. The cell state $c_{t}$ is obtained by

(4) $$c_{t}=f_{t}⊙c_{t-1}+i_{t}⊙g_{t},$$

where ⊙ denotes the Hadamard product. The hidden state $h_{t}$ is obtained by

(5) $$h_{t}=o_{t}⊙\sigma c_{t},$$

where σ denotes the state activation function of the LSTM block.

It is popular to use two well-known techniques, principal components analysis and t-sne, with deep learning. We apply these methods to process raw CAN bus data to train the LSTM network.

### 4.3. Our Method with Boosting

Ensemble learning is a machine learning technique, which is used to combine multiple methods and to get better performance than that of a particular method. The proposed boosting method combines seven state-of-the-art machine learning methods. The seven machine learning methods are support vector machine (SVM) [48,49], *k* nearest neighbour (KNN) [50], random forest (RF) [43,51], naive Bayes [52], discriminant analysis [53], adaptive boosting (AdaBoost) [54], and Long Short-Term Memory (LSTM)  [34]. The proposed boosting method is in Algorithm 2.
**Algorithm 2** The Proposed Boosting Method**Input:** Training data $D=\left\{\left(x_{1},y_{1}\right),\ldots ,\left(x_{n},y_{n}\right)\right\}$ where $x_{i}\in R^{m}$, $y_{i}=0,1$.**Output:** Final strong classifier H(x). 1: Initialize weights $w_{1,i}=\frac{1}{2c},\frac{1}{2(n-c)}$ for safe samples and unsafe samples, respectively, where *c* is the number of safe samples. 2: **for** t = 1 to U **do** 3:  Normalize the weights, $w_{t,i}=\frac{w_{t,i}}{\sum_{j=1}^{n}w_{t,j}}$. 4:  Train all *g* weak classifiers, $\left\{l_{1}\left(x\right),\ldots ,l_{g}\left(x\right)\right\}$, using the training data *D* with our time-series features. 5:  Prediction using *g* classifiers, and $h_{t}\left(x\right)$ is the classifier $l_{u}\left(x\right)$ with the highest correctly rate *a*. 6:  Update the weights: $w_{t+1,i}=w_{t,i}\times B^{1-e_{i}}$, where $e_{i}=0$, if the sample $s_{i}$ is correctly predicted, otherwise $e_{i}=1.B=\frac{a}{1-a}$. 7: **end for** 8: The boosting classifier combines the *U* classifiers: if $\sum_{t=1}^{U}\alpha_{t}h_{t}\left(x\right)\geq \frac{1}{2}\sum_{t=1}^{U}\alpha_{t}$, $H\left(x\right)=1$, otherwise $H\left(x\right)=0$.

As shown in Algorithm 2, there are *n* samples in the training data, and the dimensionality of them is equal to *m*. There are *g* weak learners used in the algorithm. In the proposed boosting method, *g* is equal to seven (see Table 3). *U* is the number of the weak classifiers which are chosen to form final strong classifier H(x). The value of *U* is a tunable parameter, and it is equal to five in our method. Each of the *g* classifiers is trained based on one particular machine learning method.

## 5. Experiments

Our experiments are conducted using two datasets: the public Warrigal dataset [55] and our own dataset provided by TransMac. The Warrigal dataset can be downloaded in http://its.acfr.usyd.edu.au/datasets/warrigal/. In the experiments, the proposed boosting method is compared with other seven popular machine learning methods: SVM, KNN, RF, Simple Bayes, Discriminant Analysis, AdaBoost, and LSTM.

### 5.1. Evaluation Metrics

The classification accuracy, sensitivity, specificity, and the Area Under Curve (AUC) are four of the most popular evaluation methods for a binary classifier. The sensitivity is the probability of detection while the specificity gives the probability of false alarm. The AUC value is equal to the probability that a randomly chosen positive example is ranked higher than a randomly chosen negative example. The sensitivity and specificity are also referred to as the True Positive Rate (TPR) and the True Negative Rate (TNR), respectively. Sensitivity and Specificity are computed using the numbers of true negatives (TN), false negatives (FN), true positives (TP), and false positives (FP):

(6) $$TPR=TP/\left(TP+FN\right),$$

(7) $$TNR=TN/\left(FP+TN\right).$$

The average accuracy is taken over ten repeated experiments to better evaluate the methods, and it is obtained by using

(8) $$\mathrm{Accuracy}=\left(TP+TN\right)/\left(TP+TN+NP+NF\right).$$

### 5.2. Experimental Setup

We use MATLAB to implement the seven methods and the proposed methods. Grid search is used to determine all the hyperparameters of our classifiers using a validation set. The predicted dependent variable is safety from 0 (safe) to 1 (unsafe). For our dataset, the training and test labels were determined by domain experts. For the other dataset, Label 1 indicates ongoing communications by the driver which can violate safety guidelines and 0 indicates the time without communications by the driver. In our dataset, there are 507 unsafe cases and 5944 safety cases. In the Warrigal dataset, there are 17,716 unsafe cases and 205,722 safety cases.

To clearly show the comparison of all methods, the experiments on each dataset are divided into two parts. In the first part, in order to demonstrate that the proposed boosting method can outperform other machine learning methods (see Table 3), all methods are trained using the raw data without our feature extraction method (see Algorithm 1). In the second part, to determine whether our feature extraction method can be used to improve the performance of machine learning methods, our feature extraction method is used to compute time-series features, and data with these time-series features are used to train the methods. By comparing the accuracies with the two sets of experiments, it is shown that the performance of methods is improved using our feature extraction method. To avoid the overfitting issue of machine learning, in both sets of the experiments, there are two scales, 70% of the dataset and 90% of the dataset, of the training set. The samples in the training set are randomly extracted from the whole dataset.

### 5.3. Safety Classification on the Warrigal Dataset

The Warrigal dataset [55] is a large dataset collected with the interactions of large trucks and smaller vehicles, i.e., thirteen vehicles in a large quarry-type environment. The data contains vehicle state information (like positions, speeds, and heading) and information on vehicle-to-vehicle communications. Due to the large size of the Warrigal dataset, when the dataset is published, the dataset is divided into many subsets with one subset for data recorded in a day. Because of the large size of the dataset and limited computational resources, only one subset (data recorded on 1st February 2009 which is just the first day in the dataset) picked from the data is used for our experiments. There are 223,438 samples with twelve features in the subset. In the dataset, there is no label for safety predictions. We consider constant communication through wireless devices as distractions which could potentially lead to unsafe driving behavior. Samples recorded during constant verbal communication are labeled as potentially unsafe (or inattentive) while the other samples are labeled safe (or attentive). More specifically, Label 1 indicates ongoing communications by the driver which can violate safety guidelines and 0 indicates driving without wireless communication engaged. 17,716 samples are labeled potentially unsafe and 205,722 are labeled safe.

The comparison among prediction models trained with the raw features of the Warrigal dataset is shown in Table 5. In terms of classification accuracy, the proposed method outperforms the other seven methods by 1.5% and 1.1%, with 70% and 90% of the dataset for training respectively. The novel method also gets the highest value of AUC. We further demonstrate the effectiveness of our feature extraction method. The performance of the models trained with our features is shown in Table 6. As observed in Table 5 and Table 6, the proposed boosting method outperforms the other seven state-of-the-art approaches whether or not our feature extraction method is used. In addition, comparison with the accuracies in Table 5 and Table 6 indicates that the performance of all machine learning methods is improved using our feature extraction method. Our method with feature extraction outperforms other methods using raw features by 2.7% and 3.2%, with 70% and 90% of all samples randomly selected for training respectively.

### 5.4. Safety Classification with Our Dataset

The results from the previous experiments show that our method improves the performance of classification. We further apply our methods to a real-world problem in the industry with a public bus company in Macao called TransMac. Given the fact that there is no real-world public dataset with labels for safety classification, the experiments are conducted using a dataset built with data collected from TransMac.

In this subsection, the experimental setup follows that of the previous subsection using a different dataset with the first set of experiments with feature extraction and the second set without. The performance comparison of the methods can be found in Table 7 and Table 8. As shown in the two tables, our boosting method outperforms the other methods in all cases. It is shown that the performance of all methods is improved using our features extraction method. Our boosting method with our feature extraction method can outperform other methods using raw features by 5.9% and 5.5%, with 70% and 90% of the whole dataset randomly selected for training respectively.

### 5.5. Further Evaluation

The CAN-bus data is time-series data. In order to see if predictions can still be accurate when training is done at a very different time period, we conduct another set of experiments to further evaluate the performance of our method. In this further experiment, the samples of the training set and the test set are collected at different times. As our real-world dataset was collected in four different periods, samples are therefore grouped into four subsets corresponding to the respective time periods. A four-fold cross-validation is conducted with these four subsets and the average classification accuracies of different methods are compared as shown in Table 9. Like Table 7 and Table 8, it is also found that the proposed method achieves the highest accuracy shown in the above table with or without our features. The boosting methods tend to be more expensive during training. We summarize the training time required for the methods in Table 10.

It is observed in our experiments that the performance does not improve when the number of weak learners in the final strong classifier, *U*, is larger than 5 although the total number of base learners built with state-of-the-art classification techniques is seven in our heterogeneous ensemble method. The empirical result verifying this is shown in Table 11.

## 6. Conclusions

Automation to determine whether certain driving behavior of drivers on public transportation can be considered safe on the road using A. I. or machine learning techniques has become a possibility recently. However, the industrial need for a high classification performance cannot be satisfied using existing methods using computer vision as the misclassification rates are too high with existing methods. Due to the high misclassification rates, it makes it hard to compare and to evaluate the performance of drivers on public transportation.

Our goal is to build a practical and accurate method for road safety predictions that automatically determine if the driving behavior is safe on public transportation. In this paper, our main contributions include (1) a novel feature extraction method because of the lack of informative features in the data, (2) a novel boosting method for driving behavior classification (safety or not) to combine advantages of deep learning and traditional statistical learning methods with much improved performance, and (3) evaluating methods using real-world data to provide accurate evaluations from labels from experts in the public transportation industry for the first time. The experiments show that the proposed boosting method with the proposed features outperforms seven other popular methods on the real-world dataset by 5.9% and 5.5%.

## Figures and Tables

**Table 1:** Features in our dataset to evaluate road safety.

Feature Name	Meaning	Used to Train our Method?
LOGID	Bus identifier	No
GPSDATE TIME	Time	No
VELOCITY	Instantaneous speed	Yes
MILEAGE	GPS mileage	Yes
TOTAL MILEAGE	Total mileage	Yes
FRONT PRESSURE	Front pressure	Yes
REAR PRESSURE	Rear pressure	Yes
ENGINESPEED	Engine speed	Yes
ENGINETEMP	Engine temperature	Yes
CARSWITCH	Switches of the bus	No
CARLIGHT STATE	Switches of light	No
CANALARM STATE	Switches of alarm	No
CREATETIME	Time	No
GPS VELOCITY	Instantaneous speed	Yes
DRIVERID	Driver identifier	No
LONGITUDE	Longitude	Yes
LATITUDE	Latitude	Yes
DIRECTION	Turn	Yes
STATIONID	Station identifier	No
ROUTEID	Route identifier	No
BUSSTATE	Bus status	No
ALARM STATE	Alarm light status	No
STATION MILEAGE	Mileage each station	Yes
UPDOWN	Up and down	No

**Table 2:** Descriptive statistics on features used for training models.

Feature Name	Mean	Std. Dev.	Median
VELOCITY	147.9	159.9	90
MILEAGE	65,220,323.4	12,868,396.8	77,710,500
TOTAL MILEAGE	66,584,813.3	15,653,632.7	81,771,740
FRONT PRESSURE	843.4	71.7	852
REAR PRESSURE	794.4	32.7	796
ENGINESPEED	976.5	342.6	803
ENGINETEMP	81.6	7.1	83
GPS VELOCITY	141.2	152.8	90
LONGITUDE	113.5	0.0066	113.5
LATITUDE	22.1	0.0195	22.1
DIRECTION	197.3	104.8	195
STATION MILEAGE	346.7	589.3	170

**Table 3:** Seven state-of-the-art machine learning methods used in the proposed boosting method.

Machine Learning Methods
Support Vector Machine (SVM)
*k* Nearest Neighbour (KNN)
Random Forest (RF)
Naive Bayes
Discriminant Analysis
Adaptive Boosting (AdaBoost)
Long Short-Term Memory (LSTM)

**Table 4:** Four components of the LSTM network to avoid the vanishing gradient problem in the RNN.

Components	Purposes
Input gate	Preprocess the input data
Output gate	Updata the output hidden state
Forget gate	Reset (forget) the input data
Cell candidate	Update the output cell state

**Table 5:** The comparison among eight methods on the Warrigal dataset without our time-series features. The proposed method gets the highest values of accuracy and AUC.

Classifying the Warrigal Dataset WITHOUT Our Features								
**Methods**	**70% Percent Data for Training**				**90% Percent Data for Training**			
	**Accu. (%)**	**AUC**	**Specificity**	**Sensitivity**	**Accu. (%)**	**AUC**	**Specificity**	**Sensitivity**
Our Method	92.9	0.923	0.932	0.912	93.7	0.931	0.939	0.922
AdaBoost	79.5	0.761	0.832	0.608	83.5	0.798	0.871	0.652
Simple Bayes	77.3	0.742	0.825	0.513	80.1	0.764	0.857	0.522
Discriminant Analysis	77.1	0.728	0.815	0.551	82.5	0.776	0.869	0.607
KNN	83.2	0.733	0.873	0.628	84.3	0.745	0.886	0.628
RF	90.7	0.903	0.923	0.828	91.8	0.910	0.935	0.830
SVM	75.9	0.756	0.722	0.896	82.3	0.815	0.796	0.906
LSTM	91.4	0.818	0.938	0.791	92.6	0.822	0.951	0.801

**Table 6:** A comparison among eight methods on the Warrigal dataset with our feature extraction method. The comparison between the values of accuracy and AUC in Table 5 and this table shows that the performance of all machine learning methods is improved with our feature extraction method.

Classifying the Warrigal Dataset WITH Our Features								
**Methods**	**70% Percent Data for Training**				**90% Percent Data for Training**			
	**Accu. (%)**	**AUC**	**Specificity**	**Sensitivity**	**Accu. (%)**	**AUC**	**Specificity**	**Sensitivity**
Our Method	94.1	0.944	0.943	0.927	95.8	0.952	0.962	0.937
AdaBoost	80.7	0.781	0.833	0.674	84.3	0.820	0.868	0.718
Simple Bayes	80.2	0.759	0.828	0.669	82.7	0.804	0.858	0.672
Discriminant Analysis	79.5	0.776	0.814	0.697	82.9	0.808	0.848	0.731
KNN	83.5	0.751	0.854	0.737	87.4	0.782	0.898	0.752
RF	91.3	0.913	0.919	0.879	93.9	0.937	0.946	0.903
SVM	78.2	0.755	0.746	0.907	82.8	0.819	0.801	0.907
LSTM	92.7	0.825	0.941	0.853	94.8	0.901	0.961	0.883

**Table 7:** The comparison among eight methods on our real-world dataset without the proposed feature extraction method. In terms of classification accuracy or AUC, our boosting method outperforms the other state-of-the-art methods in all cases.

Classifying the New Real-World Dataset WITHOUT Our Features								
**Methods**	**70% Percent Data for Training**				**90% Percent Data for Training**			
	**Accu. (%)**	**AUC**	**Specificity**	**Sensitivity**	**Accu. (%)**	**AUC**	**Specificity**	**Sensitivity**
Our Method	91.2	0.870	0.921	0.838	92.9	0.881	0.940	0.844
AdaBoost	78.1	0.740	0.808	0.574	80.0	0.753	0.827	0.592
Simple Bayes	62.7	0.586	0.643	0.521	62.0	0.577	0.634	0.515
Discriminant Analysis	58.4	0.539	0.589	0.543	60.0	0.564	0.604	0.568
KNN	72.1	0.648	0.726	0.579	75.2	0.685	0.769	0.619
RF	89.7	0.816	0.924	0.696	91.2	0.804	0.936	0.727
SVM	57.7	0.558	0.532	0.852	61.1	0.598	0.567	0.873
LSTM	77.3	0.757	0.786	0.676	85.2	0.790	0.873	0.694

**Table 8:** The comparison among eight methods on our real-world dataset with the proposed feature extraction method. After comparing with the values of classification accuracy and AUC in Table 7, it shows that the performance of any method is improved using our feature extraction method.

Classifying the New Real-World Dataset WITH Our Features								
**Methods**	**70% Percent Data for Training**				**90% Percent Data for Training**			
	**Accu. (%)**	**AUC**	**Specificity**	**Sensitivity**	**Accu. (%)**	**AUC**	**Specificity**	**Sensitivity**
Our Method	95.6	0.947	0.960	0.921	96.7	0.969	0.968	0.957
AdaBoost	83.7	0.781	0.857	0.685	85.1	0.802	0.867	0.731
Simple Bayes	66.8	0.651	0.674	0.622	72.1	0.702	0.724	0.692
Discriminant Analysis	77.8	0.698	0.794	0.651	80.1	0.732	0.816	0.686
KNN	77.2	0.716	0.789	0.640	77.6	0.727	0.790	0.669
RF	93.9	0.903	0.958	0.790	94.1	0.904	0.956	0.827
SVM	77.9	0.689	0.753	0.857	80.4	0.732	0.781	0.881
LSTM	75.1	0.734	0.765	0.642	80.3	0.767	0.820	0.675

**Table 9:** Classification accuracies of the eight methods using our real-world dataset. In this experiment for this table, the samples of the training set and test set are collected in four different periods; samples are therefore grouped into four subsets corresponding to the respective time periods.

On Our Real-World Dataset		
**Methods**	**With Our Features**	**Without Our Features**
Our Method	86.4%	68.7%
AdaBoost	69.3%	66.4%
Simple Bayes	69.4%	59.9%
Discriminant Analysis	62.3%	58.7%
KNN	71.9%	56.9%
RF	65.6%	61.1%
SVM	84.1%	62.0%
LSTM	82.3%	63.9%

**Table 10:** A summary of the training time required for the methods compared in seconds.

	On Our New Dataset				On the Warrigal Dataset			
	With Our Features		Without Our Features		With Our Features		Without Our Features	
Scales of Training Set	70%	90%	70%	90%	70%	90%	70%	90%
Our Method	12.8	17.4	7.5	10.6	42.1k	62.9k	21.7k	34.5k
AdaBoost	2.7	3.3	1.9	2.2	13.3k	17.2k	9.72k	14.5k
Simple Bayes	0.11	0.13	0.04	0.05	6.6k	8.3k	4.52k	6.1k
Discriminant Analysis	0.08	0.10	0.04	0.07	1.7k	2.1k	1.1k	1.5k
KNN	0.07	0.12	0.06	0.08	5.4k	7.2k	3.2k	5.1k
RF	0.35	0.49	0.31	0.35	2.9k	3.6k	2.1k	2.4k
SVM	2.1	2.8	1.5	2.6	12.6k	15.4k	8.7k	12.5k
LSTM	1.9	2.3	1.6	2.0	11.7k	14.2k	7.1k	10.5k

**Table 11:** A summary of the classification accuracy of the proposed boosting method with different values of parameter *U*.

Values of *U*	2	3	4	5	6
Methods Trained with 70% Examples	91.2%	93.5%	93.9%	94.1%	94.0%
Methods Trained with 90% Examples	92.7%	93.9%	95.6%	95.8%	95.2%
